# Insights into the role of diet and dietary flavanols in cognitive aging: results of a randomized controlled trial

**DOI:** 10.1038/s41598-021-83370-2

**Published:** 2021-02-15

**Authors:** Richard P. Sloan, Melanie Wall, Lok-Kin Yeung, Tianshu Feng, Xinyang Feng, Frank Provenzano, Hagen Schroeter, Vincenzo Lauriola, Adam M. Brickman, Scott A. Small

**Affiliations:** 1 Division of Behavioral Medicine, Department of Psychiatry, Columbia University Irving Medical Center, 622 West 168th St., New York, NY 10032 USA; 2 New York State Psychiatric Institute, 1050 Riverside Drive, New York, NY 10032 USA; 3 Department of Neurology, Vagelos College of Physicians and Surgeons, Columbia University, 622 West 168th St., New York, NY 10032 USA; 4 Taub Institute for Research On Alzheimer’s Disease and the Aging Brain, Vagelos College of Physicians and Surgeons, Columbia University, 622 West 168th St., New York, NY 10032 USA; 5 Mars Inc., 6885 Elm St, McLean, VA 22101 USA

**Keywords:** Neuroscience, Cognitive ageing, Cognitive neuroscience

## Abstract

With the world's population aging, age-related memory decline is an impending cognitive epidemic. Assessing the impact of diet on cognitive aging, we conducted a controlled, randomized, parallel-arm dietary intervention with 211 healthy adults (50–75 years) investigating effects of either a placebo or 260, 510 and 770 mg/day of cocoa flavanols for 12-weeks followed by 8-weeks washout. The primary outcome was a newly-developed object-recognition task localized to the hippocampus’ dentate gyrus. Secondary outcomes included a hippocampal-dependent list-learning task and a prefrontal cortex-dependent list-sorting task. The alternative Healthy Eating Index and a biomarker of flavanol intake (gVLM) were measured. In an MRI substudy, hippocampal cerebral blood volume was mapped. Object-recognition and list-sorting performance did not correlate with baseline diet quality and did not improve after flavanol intake. However, the hippocampal-dependent list-learning performance was directly associated with baseline diet quality and improved after flavanol intake, particularly in participants in the bottom tertile of baseline diet quality. In the imaging substudy, a region-of-interest analysis was negative but a voxel-based-analysis suggested that dietary flavanols target the dentate gyrus. While replication is needed, these findings suggest that diet in general, and dietary flavanols in particular, may be associated with memory function of the aging hippocampus and normal cognitive decline.

## Introduction

As the average age of populations increases globally, so too do the considerable societal and personal costs associated with a decline in cognitive performance during normal cognitive aging. Consequently, research in this area increasingly focuses on the development of life-style and dietary approaches aimed at preventing or delaying the onset of age-related cognitive decline. The work described here lies at the intersection of two main areas of interest and scientific inquiry, namely the identification and investigation of specific brain regions causally linked to cognitive aging as well as the impact of diet on cognitive performance and its application as a potential means for maintaining cognitive health into old age. In this context, data from epidemiological studies as well as dietary intervention studies support the notion that dietary status generally, and the intake of dietary flavanols, in particular, may attenuate cognitive aging and enhance various fuctional measures of cognitive performance^1,2^. Flavanols are bioactive food constituents and dietary sources include tea, cocoa, pome fruits, grapes and wine, and various grains, herbs, and berries.

While the physiological processes that underlie cognitive aging are still being investigated, the neuroanatomical profile of cognitive aging is currently thought to be localized to two general brain areas, the prefrontal cortex and hippocampus^3^. Nonetheless, further experimental interventions are required to validate that these two brain regions are causally linked to cognitive aging. Initial experimental data, including a video game-based intervention, which demonstrated improved performance on a prefrontal task that declines with age, strengthen the argument for a causal link between the prefrontal cortex and cognitive aging^4^.

**Figure 1:**
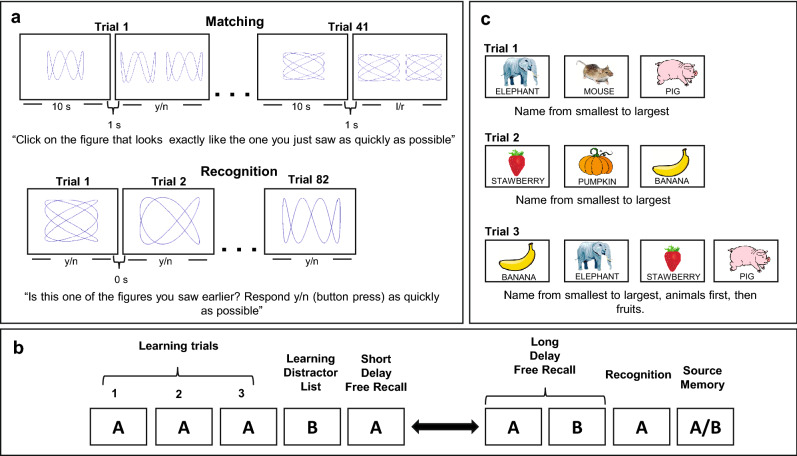
Example of the neuropsychological tests used to assess cognitive performance, including (**a**) Object-Recognition test (Modified Benton recognition task), (**b**) List-Learning memory test (Modified Rey auditory verbal learning test), and (**c**) List-Sorting task (List sorting working memory test from the NIH Toolbox Cognition Battery). Example stimuli for the ModBent (**a**) were generated in MATLAB (MathWorks, Natick, MA, version R2008a). Details of stimuli generation were described previously^8^. Stimuli displayed as examples from NIH Toolbox were publicly available clip art. We did not include stimuli from the actual tests to maintain the integrity and security of test materials, according to the ethical standards of our field. Information about the NIH Toolbox is available online (https://www.healthmeasures.net/explore-measurement-systems/nih-toolbox).

We have investigated the potential causal link between cognitive aging and functional changes in the dentate gyrus (DG), a region within the hippocampal circuit vulnerable to aging^5^. The DG has been linked to performance on "object-recognition" tasks^6^ and other investigators suggested that DG function is essential in the pattern separation of complex stimuli as they flow through the hippocampal circuit^7^. Using a new task in which a series of complex patterns were embedded in an object-recognition paradigm sufficiently difficult to probe pattern separation ability in humans^8^ (Fig. 1a), our prior proof-of-concept study showed that a smaller-scale flavanol-based dietary intervention resulted in improved performance on this task, supporting the hypothesis that age-related modulations of function in the hippocampus in general, and the DG in particular, contributes to cognitive aging^8^.

Here, we aimed to replicate our initial findings at greater scale and conducted a 20-week placebo-controlled cocoa flavanol-based clinical dietary intervention study in 211 healthy older participants. Expanding upon the scope of our previous investigation, we included a broader intake amount range, investigated the role of baseline diet quality on cognitive performance, and studied the persistence of flavanol intake-related effect after consumption was discontinued. The aim of the trial was to investigate whether or not flavanol consumption improves performance on a standard hippocampal-dependent declarative memory task (Fig. 1b) and a prefrontal cortex-dependent list-sorting task (Fig. 1c).

## Results

### Study participants

**Figure 2:**
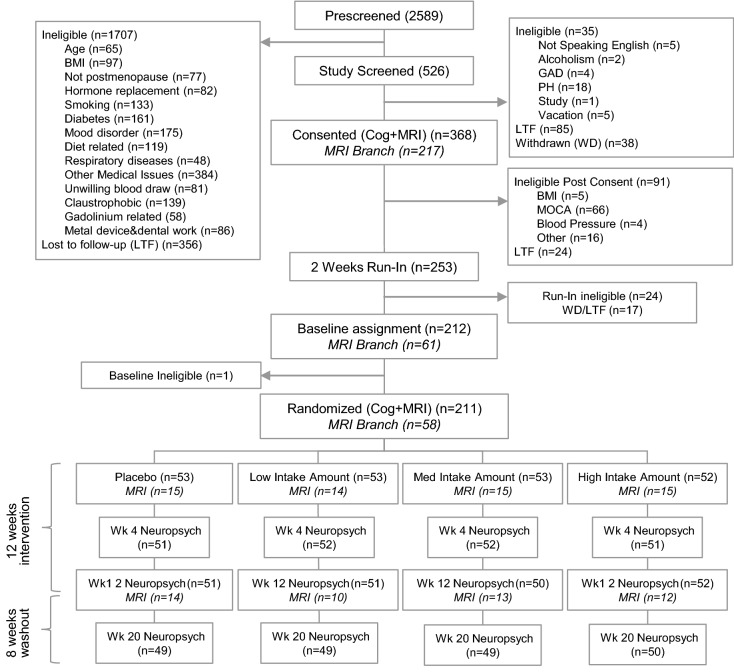
CONSORT diagram.

Data collection began on Jan. 13, 2016 and ended on Nov. 21, 2018. A total of 2589 potential participants were screened, of whom 526 initially were eligible. 368 provided informed consent and 212 successfully completed the run-in phase. 211 (120 women, 91 men, 61.99 ± 6.44 years old) were randomized in the main study stratified by age and sex, and 58 were randomly selected for the MRI substudy based on eligibility and designed to evenly space the MRI data collection across the full three years of the main study enrollment. Participants were randomly assigned to four different levels of flavanol intake: placebo/0 mg, n = 53; low intake, n = 53; middle intake, n = 53; high intake, n = 52. In the substudy, 15, 14, 14, and 14 participants were randomized to these four intake conditions respectively. Further details are presented in the CONSORT diagram (Fig. 2).

### Participant retention and adherence to the treatment protocol

204 participants completed the main study assessments after 12 weeks of dietary flavanol intake (204/211 = 97% retention rate) and 49 completed the MRI substudy. Demographic characteristics of the sample appear in Table 1. There were no serious adverse events attributable to the trial. In the MRI substudy, one participant experienced a vasovagal reaction to insertion of the IV line and another experienced nausea.

**Table 1:** Demographics characteristics and cognitive and MRI measurements in study population at baseline.

Variables	Total Samples (n = 211)		Treatment Groups								*p* value^a^	Standardized Mean Differences between groups^b^
			Placebo group (n = 53)		Low flavanol intake group (n = 53)		Medium flavanol intake group (n = 53)		High flavanol intake group (n = 53)			
	n	Mean (SD) or %	n	Mean (SD) or %	n	Mean (SD) or %	n	Mean (SD) or %	n	Mean (SD) or %		
**Demographics**												
Age (range 50–75)	211	61.99 (6.44)	53	62.35 (6.70)	53	61.88 (6.10)	53	61.65 (6.69)	52	62.07 (6.41)	0.955	0.059
Gender												
Male	91	43.10%	22	41.50%	23	43.40%	23	43.40%	23	44.20%	0.994	0.027
Female	120	56.90%	31	58.50%	30	56.60%	30	56.60%	29	55.80%		
Education Category												
Bachelor's or Less	116	55.00%	26	49.10%	30	56.60%	25	47.20%	35	67.30%	0.151	0.227
More than Bachelor's	95	45.00%	27	50.90%	23	43.40%	28	52.80%	17	32.70%		
**Cognitive Measures**												
Baseline Novel Object-Recognition	210	2680.6 (1309.4)	53	2807.98 (1733.36)	53	2579.94 (1026.18)	53	2603.65 (999.31)	53	2732.79 (1367.96)	0.785	0.104
Baseline List-Learning	208	38.96 (8.45)	53	38.91 (8.45)	52	37.25 (8.19)	52	39.75 (7.38)	51	39.96 (9.62)	0.349	0.177
Baseline List-Sorting	208	54.86 (8.94)	53	52.89 (8.85)	52	56.81 (8.91)	52	54.60 (9.15)	51	55.18 (8.65)	0.16	0.23
**MRI Measures**												
Baseline DG-CBV	57	2.15 (0.69)	15	2.44 (0.81)	14	2.17 (0.69)	14	2.11 (0.47)	14	1.85 (0.67)	0.153	0.442
**Other relevant variables**												
aHEI	211	66.36 (12.0)	53	65.09 (11.44)	53	68.78 (10.82)	53	64.83 (14.3)	52	66.74 (10.84)	0.299	0.188
gVLM	208	30.44(55.84)	51	19.70 (24.23)	53	20.98 (34.28)	53	44.32 (72.4)	51	36.59 (72.06)	0.0617	0.267

^a^Baseline differences are assessed using F-tests (3 df) for continuous measures and chi-square test (3 df) for dichotomous measures.

^b^Standardized absolute mean differences is calculated as the average absolute difference between all 6 comparisons of treatment groups divided by the overall standard deviation. Values > 0.25 are considered to be non-trivial imbalance due to chance.

aHEI, alternative Healthy Eating Index; gVLM, 5-(3ʹ,4ʹ-dihydroxyphenyl)-γ-valerolactone metabolites; DG-CBV, dentate gyrus-cerebral blood volume.

**Figure 3:**
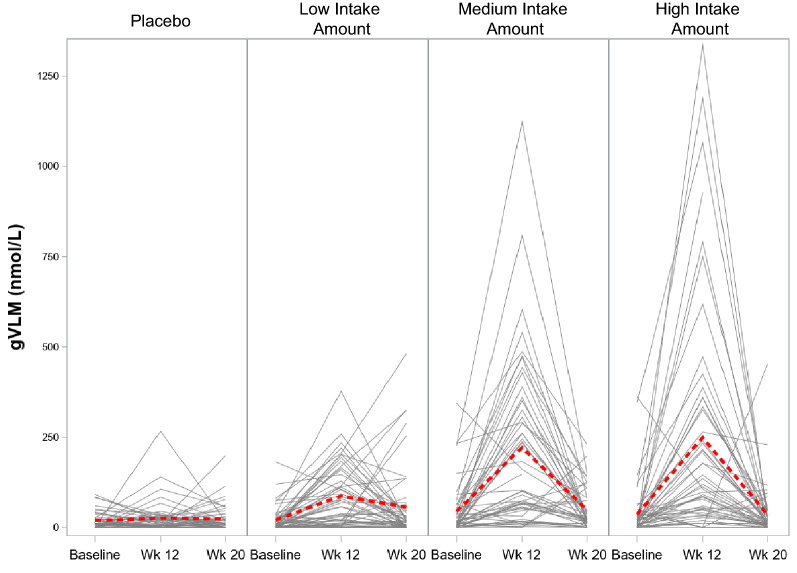
Concentration of 5-(3ʹ,4ʹ-dihydroxyphenyl)-γ-valerolactone metabolites (gVLM) in plasma at baseline, 12 weeks after daily intake of placebo and flavanols at a low (260 mg), medium (510 mg) and high (770 mg) intake level, and at 20 weeks after washout (8 weeks). Data are presented as the individual concentration of gVLM for each volunteer (black lines) and as the mean (red dashed line) over time.

Blood tests at baseline and after 12 and 20 weeks revealed an intake amount-dependent increase then decrease in gVLM, a biomarker of dietary flavanol intake (Fig. 3), demonstrating the bioavailability of flavanols from the test materials and adherence to the interventions. 8 weeks after the end of the capsule intake period, gVLM levels had returned to baseline levels.

Capsule count data were consistent with the gVLM assessment. Mean capsules consumed/day were 3.99 ± 1.11, 3.98 ± 0.55, 3.88 ± 0.29, and 3.85 ± 0.32 out of the required 4/day for the placebo, low, medium, and high flavanol groups respectively. Adherence was not different across treatment groups.

As Table 1 indicates, covariate balance between randomized treatment groups was achieved for demographic characteristics and baseline cognitive measures (all standardized mean differences (SMD) < 0.25). Non-trivial imbalance due to chance randomization occurred between treatment groups on baseline DG-CBV (SMD = 0.44) as is common given the smaller sample sizes selected to the MRI substudy. All analyses of treatment effects controlled for baseline differences, sex, age, and education.

### Object-recognition performance

Baseline performance on the object-recognition task did not correlate with aHEI (r = 0.027, *p* = 0.694), gVLM (r = − 0.059, *p* = 0.397), and surprisingly, it did not correlate with age (r = 0.055, *p* = 0.428). There was no significant effect of 12 weeks of flavanol consumption on performance on this task (*p* = 0.441, Table 2).

**Table 2:** Mixed effects model results between treatment group effects at week 12 (end of treatment), controlling for basline score, sex, age and education.

Between Groups	Low flavanol intake Vs. Placebo				Medium flavanol intake Vs. Placebo				High flavanol intake Vs. Placebo				Test of Intake amount Response Effect
Measure	Adj Mean Diff	SE	Cohen's d Effect size^a^	p^b^	Adj Mean Diff	SE	Cohen's d Effect size^a^	p^b^	Adj Mean Diff	SE	Cohen's d Effect size^a^	p^b^	p^c^
**Neurocognitive Measures**													
Novel Object-Recognition	− 8.917	154.92	− 0.007	0.954	− 147.065	155.426	− 0.112	0.345	21.693	156.072	0.017	0.890	0.441
List-Learning	0.592	1.178	0.07	0.616	0.996	1.187	0.118	0.402	2.024	1.184	0.239	0.088	**0.042**
List-Sorting	0.416	1.444	0.047	0.773	− 0.16	1.45	− 0.018	0.912	− 1.245	1.444	− 0.139	0.389	0.172
**MRI Measure**													
CBV	− 0.084	0.253	− 0.122	0.743	− 0.165	0.238	− 0.24	0.493	0.169	0.262	0.246	0.524	0.307

Bold value indicates *p* < 0.05.

^a^Cohen’s d effect size calculated taking Adj Mean Diff divided by baseline measures standard deviation.

^b^Two-sided *p* value for t-test of treatment at each intake amount compared to placebo from longitudinal mixed effects model.

^c^One-sided p-vale for t-test of linear trend in treatment effects across groups from longitudinal mixed effects models.

Adj Mean Diff, adjusted mean difference; CBV, Cerebral Blood Volume.

Post-hoc analysis revealed technical issues in the implementation of the object-recognition task: only one third of subjects performed the task at better than chance, based on the assumption that the percent accuracy out of the 82 trials was expected to randomly fall with 95% certainty in the range from 40.2 to 59.7%. Thus, the failure to support the primary hypothesis may have been due to problems with the psychometric properties of the outcome measure.

### List-learning performance

We also tested the effect of flavanol intake on a hippocampal-dependent ‘list-learning’ task. At baseline, performance on this task positively correlated with diet quality and habitual flavanol intake, as measured by the aHEI (r = 0.141, p = 0.043) and the blood-based flavanol biomarker gVLM (r = 0.138, *p* = 0.049), respectively. It also correlated with younger age (r = − 0.215, *p* = 0.002).

**Figure 4:**
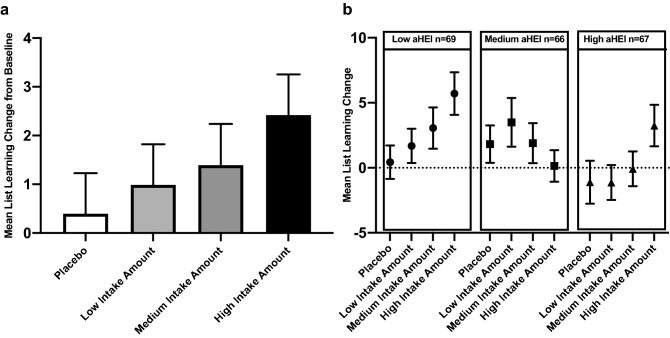
Changes in List-Learning memory test before and 12 weeks after daily intake of placebo and flavanols at a low (260 mg), medium (510 mg) and high (770 mg) intake level. Data are expressed as mean ± SE of all volunteers (**a**) and divided in tertiles according to diet quality assessed with the alternative Healthy Eating Index (aHEI; **b**).

Controlling for age, sex, level of education, and baseline test performance, there was a significant intake level-dependent treatment effect compared to placebo (one-tailed test of trend, *p* = 0.042; Table 2, Fig. 4a) after 12 weeks of intervention. Furthermore, the effect of dietary flavanols on improvement in the task was associated with baseline aHEI (Table 3). A marginally significant treatment by aHEI interaction was observed (F(6,186) = 1.87, *p* = 0.088, Table 3). While the flavanol effect was observed across the cohort, a post-hoc exploration revealed that the effect was driven primarily by participants with low scores of baseline aHEI (Fig. 4b). For those in the bottom tertile of the aHEI at baseline, the effect of high flavanol intake on list learning was significantly greater than placebo (Cohen’s d = 0.623, *p* = 0.012; Table 3).

**Table 3:** Test of differential effects between treatment groups on the List-Learning task at week 12 at different tertiles divided according to alternative Healthy Eating Index (aHEI), controlling for baseline list learning, sex, age, and education.

Between Groups	Low aHEI at Baseline				Medium aHEI at Baseline				High aHEI at Baseline			
Tests between Intake amounts	Adj Mean Diff	SE	Cohen's d Effect size^a^	p^b^	Adj Mean Diff	SE	Cohen's d Effect size^a^	p^b^	Adj Mean Diff	SE	Cohen's d Effect size^a^	p^b^
Low flavanol intake vs Placebo	1.251	1.846	0.148	0.499	1.673	2.366	0.198	0.480	− 0.022	2.1	− 0.003	0.992
Medium flavanol intake vs Placebo	2.621	2.047	0.31	0.202	0.078	2.109	0.009	0.971	1.028	2.11	0.122	0.627
High flavanol intake vs Placebo	5.268	2.085	0.623	**0.012**	− 1.676	1.891	− 0.198	0.377	4.355	2.29	0.515	0.059
Trend Test across intake amounts				**0.011**				0.271				0.051
Interaction test (aHEI vs intake amount)				F-test (6,186) = 1.87, *p* = 0.088								

Bold value indicates *p* < 0.05.

^a^Cohen’s d effect size calculated taking Adj Mean Diff divided by baseline measures standard deviation.

^b^Two-sided *p* value for t-test of treatment at each intake amount compared to placebo from longitudinal mixed effects model.

Adj Mean Diff, adjusted mean difference;

The interaction between baseline gVLM levels and treatment was not statistically significant (F(6, 183) = 1.66, *p* = 0.134), hence further interpretation of differential treatment effects by baseline gVLM should be considered exploratory. After controlling for baseline list learning, sex, age, and education there was no significant association with baseline gVLM levels (*p* = 0.831).

Finally, 8 weeks after cessation of flavanol consumption, there were no effects on list learning of the intervention compared with placebo at any flavanol intake level, reflecting a return to baseline performance (one-tailed test of trend across flavanol intake at 8 weeks after cessation, *p* = 0.485).

### List-sorting performance

We also assessed a list-sorting task that functionally localizes to the prefrontal cortex. Baseline performance on this task trended towards an inverse relationship with the aHEI (r = − 0.13, *p* = 0.07) but was not associated with gVLM (r = 0.01, *p* = 0.85). There was no effect 12 weeks of flavanol consumption on this task (*p* = 0.172, Table 2) and there were no observed interactions with baseline aHEI or gVLM.

### CBV-fMRI substudy

**Figure 5:**
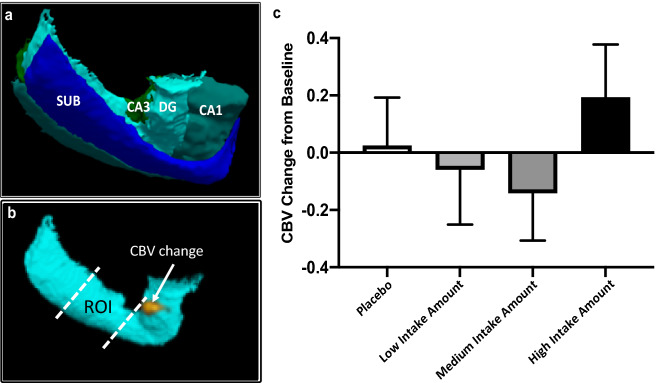
(**a**) Hippocampal sub-regions. (**b**) Predetermined region-of-interest (ROI) in the Dentate Gyrus used to evaluate the effect of flavanol consumption by Cerebral Blood Volume (CBV)-fMRI. (**c**) Changes in CBV-fMRI before and 12 weeks after daily intake of placebo and flavanols at a low (260 mg), medium (510 mg) and high (770 mg) intake level. Data are expressed as mean ± SE adjusted by baseline values.

The DG, together with other regions of the hippocampus, extends for over 5 cm in its long axis (Fig. 5a). Guided by our previous study, we established a predetermined region-of-interest (ROI) in the middle of the DG (Fig. 5b), which we used to evaluate the effect of flavanol consumption by CBV-fMRI. There was no effect of 12 weeks of flavanol consumption on CBV-fMRI (*p* = 0.307; Table 2, Fig. 5c). However, the effect size for high intake versus placebo for CBV-fMRI (Cohen’s d = 0.246) was similar to that found for the list learning task and a significant effect was observed when not controlling for baseline performance (Supplementary Fig. 1). The sample size in the MRI substudy was originally based on power considerations assuming a much larger effect size (expected Cohen’s d = 1.9) based our previous study^8^.

We then performed a voxel-based analysis to localize the flavanol effect in the hippocampus. Flavanol consumption increased CBV that localized to the anterior head of the DG, at the outer boundaries of the ROI (Fig. 5b), but in no other hippocampal regions.

## Discussion

This study was designed to replicate and extend the findings from our previous study into a larger sample, investigate a range of flavanol intake amounts, and assess the persistence of effects on cognitive function related to multiple brain regions.

Flavanol intake did not improve performance on the object-recognition task, the study's primary endpoint, failing to replicate our previous findings. This failure to replicate tenably reflects technical limitations of the newly developed object-recognition task. First, the task was designed to be difficult enough to stress the DG’s purported function in pattern separation. However, *post-hoc* analysis suggested that the task, originally developed with younger subjects, is so difficult that the majority of participants performed no better than chance, which likely contributed to the failure to replicate. Challenges related to the level of difficulty of a given cognitive task, especially when investigating the impact on cognition of nutrition- and life-syle modifications in generally healthy individuals have been raised and discussed before^1,9^. The level of task difficulty seems to be a critical factor, not just when choosing or designing a cognitive task for testing a specific hypothesis, but also when interpreting outcomes and observed effects sizes. Thus, this notion is not exclusively relevant for discussing the object-recognition task used here, but also applies the other cognitive tasks deployed here and elsewhere. It can be anticipated that future deployment of difficulty-ranked cognitive assessments will help address this challenge.

Second, the standard list-learning memory task, a secondary outcome, is intuitively easy to understand and perform. Our data demonstrate that list learning-task performance was improved by the dietary flavanol intervention. It is noteworthy that the effect size for the observed improvements in this cohort of generally healthy individuals seems to be directly related to habitual diet quality. Individuals with lower aHEI score more likely experienced greater treatment effects. Our data also demonstrate that shifting flavanol intake back to reflect habitual intake levels at baseline, causes an attenuation of memory performance gains within 8 weeks of intervention cessation. This finding provides novel insights into the dynamics and functional plasticity of list-lerning task performance in the context of nutrition, supporting the notion that this hippocampal-dependent task is sensitive to relatively short-term dietary modifications.

While epidemiological studies as well as dietary interventions have previously demonstrated associations between flavanol intake and cognitive performance^1,2,9^, detailed assessements of diet quality and habitual flavanols intake in well-controlled dietary interventions in the context of cognitive performance and cognitive aging are uncommon. Thus, our data on the link between habitual diet, baseline cognitive performance, and intervention-based effect size may help explain some of the heterogenicity related to outcomes across previous flavonoid-centric dietary interventions in the area of cognition, and they may also have utility in the context of interpretating our findings in a broader population-based context. Although at the level of an individual, the observed effect sizes were modest, especially when considered in the context of pharmaceutical treatments of disease, similar scale improvements in the context of diet and lifestyle modifications have been shown to have meaningful impact on health and disease risk reduction at the population level. For example, a small reduction in dietary salt intake can have a significant impact on coronary heart disease^10^, small reductions in systolic blood pressure are associated with a population-level reduction in heart failure events^11^, and a small fortification of cereal grains with folic acid significantly reduces the prevalence of neurodevelopment birth defects in the population^12^. These and other studies show the benefits of population-based dietary advice and public health interventions for conditions that normally occur in early or midlife^13^. This potential benefit is underscored by a comparison of the aHEI scores in our study to those from the general US public. The median aHEI of study participants was 66 (IQR 58 - 74). In NHANES, the mean aHEI was 39.0 (95% CI 38.9 39.8) in women and 35.7 (95% CI 34.9; 36.6) in men^14^, suggesting that the general US public has a poorer diet quality than the bottom tertile of participants in this study, and may thus tenably experience cognitive benefits from increasing their diet quality and flavanol intake.

This observation suggests the value of assessing diet quality to determine those individuals who may benefit from flavanol supplementation. While these findings suggest the value of comprehensive measures of diet quality and intake of specific nutrients/dietary constituents such as flavanols when investigating the effects of dietary interventions on cognition, they require further study. The outcomes of an ongoing large-scale and long-term dietary intervention trial, COSMOS^15^, may provide more conclusive answers.

In the context of neuroanatomical insights into cognitive aging, the well-established list learning performance task was not explicitly developed to assess pattern separation. Studies suggest that performance on list-learning trials of a declarative memory task depends, in part, on this computational operation^16^ and that among hippocampal regions, it differentially depends on the DG^17,18^. Further support for this interpretation comes from the voxel-based analysis of the CBV-fMRI data obtained here. Although conducted only on a small subset of participants, the DG was the only hippocampal region that increased its basal metabolism after flavanol consumption. The lack of an effect on the ROI analysis may be due to insufficient statistical power. Since pattern separation occurs on a continuum^7^, large-scale data will allow us to sort the items from the newly developed task by degree of difficulty to compare performance on ‘hard’ vs. ‘easy’ items, further refining this task. An intriguing finding is that flavanol intake improved performance on a hippocampal-dependent, but not on a prefrontal cortex task, suggesting a regional specificity of the effect.

The principal limitation of the trial was the failure of the object-recognition task to perform as expected. Designed to overcome the limitation of its predecessor, the Benton Visual Retention Task, originally designed to test patients with profound cognitive impairments^8^, the newly developed task was designed to be more challenging so as to ‘stress’ the DG among healthy elders. Our finding, showing that most participants performed no better than chance, suggests that it might be too challenging, compromising our ability to adequately test the primary hypothesis.

A second limitation was the failure of randomization to produce treatment groups with approximately equivalent levels of DG-CBV at baseline. After adjustment for these baseline levels, there was no effect of flavanols on CBV. It is impossible to know whether the failure to detect an effect was the result of this randomization failure or in fact, there is no effect on CBV.

We also considered the presence of the caffeine in our test materials (Table A, Supplementary Methods), and would argue that this does not represent a confounder in this study and its outcomes interpretation. While the effects of caffeine are well documented in the context of its transient impact on alertness, mood, arousal, and concentration, to our knowledge, sustained beneficial effects of caffeine at intake amounts at or below 25 mg per day on memory and cognitive performance have not been demonstrated by either epidemiological- or intervention studies^19,20^. Moreover, 3 preceding dietary intervention studies in the context of which caffeine levels were matched across treatment groups, clearly identified caffeine-independent effects of flavanol intake on various measures of memory and cognitive performance^8,21,22^. In addition, as we were interested in the longer-term, and not the acute effects of flavanols on memory and cognitive function, our study was designed to undertake all cognitive performance tests at least 12 h after flavanol intake, thus excluding the potential transient impact of caffeine on vasomotor function as well as on the other endpoints investigated. This is further supported by measurements of caffeine in plasma collected at each study visit^23^, which did not identify significant differences in the levels of caffeine after test material intake (at baseline: 6 ± 6 µM; at week 12: 7 ± 9 µM; at week 20: 7 ± 9 µM; average ± SD across all samples).

It is also noteworthy that this study was focused on participants with normal cognitive function, thus the generalizability of our findings is limited and cannot be extended into patient populations with clinical manifestations of dementia or cognitive dysfunction. Finally, our study is limited by the intervention- and total observation periods of 12 and 20 weeks, respectively. While ranking among the longer-term clinical dietary intverventions undertaken in the field of polyphenols/nutrition and cognition, the study duration does represent a limitation.

In conclusion, this study raises the possibility that at the population level, flavanol-based dietary interventions may have a beneficial impact on cognitive aging. Considering the increasing world-wide aging population due to the overall reduction in late life morbidities, normal age-related memory decline is now considered an impending cognitive epidemic^24^. In this context, dietary flavanols may offer meaningful benefits to cognitive health, although further studies are needed. Replication of our findings at scale, potentially through ongoing studies like COSMOS^15^, may allow for an evidence-based assessment at the population level of the utility of dietary flavanols to address the significant challenge of age-related cognitive decline in late life.

## Methods

### Design and ethics

This parallel-groups, 4-arm, placebo-controlled trial was approved by the Institutional Review Board of the New York State Psychiatric Institute in accordance with the Helsinki Declaration of 1975 as revised in 1983. Informed consent was obtained from all participants. The trial was registered with ClinicalTrials.Gov (NCT02312310).

### Study participants

Study participants were 211 healthy, sedentary, normotensive older adults, 50–75 years of age, with a BMI between 18.0 and 35.0 kg/m^2^, recruited by social media and radio ads from the metropolitan New York City area. All women were post-menopausal. Exclusion criteria included Montreal Cognitive Assessment scores < 26, current depression or anxiety symptoms (PHQ-8 score >  = 10 and/or GAD-7 score >  = 10), smoking, current or history of substance use, use of psychotropic medications, current psychiatric disorder, habitual consumers of dietary or herbal supplements including Gingko, flavonoid, and dietary herbal or plant extracts, lactose intolerance, and diabetes. MRI-related exclusion criteria appear in Supplementary Methods.

### Study protocol

Participants deemed eligible by phone screening met with a study research assistant to provide written informed consent and further determine eligibility. At the consent appointment, participants were given the opportunity to enroll in an optional MRI substudy or only in the main study. Eligible participants completed a 2-week run-in period, described below, then were scheduled for the time 0 neuropsychological testing session and two weeks later, the time 1 measurement session, during which they provided a fasting blood sample, ate a light breakfast, then completed another neuropsychological test battery, a dietary inventory, and if enrolled in the optional substudy, an MRI study (described below). After completing these tests, they were randomized to a treatment condition. After the 12-week intervention period, they returned for a post-intervention testing session and then, 8 weeks later, a final testing session. Participants in the substudy had a second MRI session immediately following the intervention period.

### Test material description

The cocoa flavanol-based intervention materials were supplied in a capsule format. Detailed information related to the test material characterization is provided in the Supplementary Methods.

### Run-in period

During this two-week period, participants were to take two placebo capsules each morning and evening. If they consumed 80% of the capsules, as assessed by pill count, they qualified for continued participation.

### Treatment interventions

Participants were randomly assigned to four levels of daily dietary flavanol intake for the 12-week intervention period: placebo (0 mg of Flavanols per day), 260 mg, 510 mg, and 770 mg of Flavanols per day (for durther details, please see Supplementary Methods). Randomization was done using computer-generated lists of random numbers via the randomly permuted block method and stratified by sex and age group (50–63 and 64–74 years) for the overall study and for the MRI substudy. The allocation ratio to receive placebo and the 3 flavanol interventions was 1:1:1:1. The allocation list was generated by MW, recruitment of participants was conducted by VL, and allocation of participants was conducted by TF.

### Blinding

All study staff as well as participants were blinded to treatment conditions, which were identified only by randomly generated identifiers.

### Laboratory testing sessions

Participants arrived at the Behavioral Medicine Laboratory at 8 am after an overnight fast. After a venous blood draw, they received a light breakfast followed by a food frequency questionnaire (see below) and neuropsychology tests. Participants in the optional substudy then had an MRI scan.

### Measurement of adherence to study protocol

Adherence to the study protocol was measured by biological and behavioral assessments. gVLM, a flavanol metabolite and validated biomarker of intake^23,25^, was measured at baseline and after the 12-week intervention. We also measured capsule counts from baseline to week 12. Capsules taken were computed as the difference between the number of capsules provided and the number remaining after the 12-week intervention.

### Treatment assessments

#### Neuropsychological tests to assess cognitive performance

Neuropsychological tests were conducted at weeks − 2, 0, 4, 12, and 20. The test session at week − 2 was administered to minimize practice effects during the active study period and was not considered in analyses. The primary outcome measure was performance on an object-recognition task, the Modified Benton Recognition Task (Fig. 1a), previously linked to DG function, and previously described^8^. A standardized list-learning memory test, the Modified Rey Auditory Verbal Learning Test (Fig. 1b) was administered as a second hippocampal-mediated measure^26^. For this test, the total number of words learned across three trials was the outcome measures. A List Sorting Working Memory Test (Fig. 1c) from the NIH Toolbox Cognition Battery also was administered^27^.

### MRI acquisition

Subjects eligible for cerebral blood volume (CBV)-fMRI scans received them at two times (weeks 0 and 12), according to eligibility criteria as previously described^8^.

### MRI processing

Whole brain CBV images were generated from the pair of pre- and post-contrast MRI images as described previously^8,28^, and based on our previous findings, a region-of-interest was identified in the body of the dentate gyrus (see Supplementary Methods).

### Blood assays

A series of 5-(3ʹ,4ʹ-dihydroxyphenyl)-γ-valerolactone metabolites (gVLM) derived from flavanol gut microbiome catabolism were measured in plasma at the Department of Nutrition at the University of California, Davis as previously described^29^.

### Dietary assessment

We used the Block 2005 food frequency questionnaire (FFQ) (NutritionQuest, Berkeley CA; (www.NutritionQuest.com) to estimate customary dietary intake. The food list for this questionnaire was developed from the NHANES III dietary recall data. The nutrient database was developed from the USDA Nutrient Database for Standard Reference. Individual portion size was measured using pictures provided by the FFQ. From these data, we computed the alternative Healthy Eating Index (aHEI)^30,31^.

### Participant compensation

Participants were paid $40 for each of the five neuropsychology testing sessions. If they consumed at least 90% of the total number of required capsules and completed all testing sessions, they received an additional $50 for a possible total of $250. Participants in the optional MRI sub-study received $70 for each of the two MRI scans.

### Statistical analyses

Demographics, baseline cognitive, and imagining measures were summarized by randomized treatment groups and differences tested using ANOVA and chi-square tests. Based on recommendation not to focus on statistical tests of baseline differences we also calculated standardized absolute mean differences as the average absolute difference between all 6 comparisons of treatment groups divided by the overall standard deviation. Values > 0.25 are considered to be non-trivial imbalance due to chance. Pearson correlation coefficients were used to examine the association between baseline healthy eating, gVLM, and cognitive and imaging measures.

The flavanol effect on the change in each outcome from baseline to 12 weeks was tested using linear mixed effects models controlling for the respective baseline measures, four categories of treatment, sex, age, and education. Regression adjusted mean within-group tests of change were estimated and tested for statistical significance from the model. The primary test used for assessing the treatment effect was the linear trend contrast from the model across: placebo, low, medium and high intake. The model for cognitive measures incorporated additional outcome measurement times at 4 weeks and 20 weeks and included categorical time (4, 12, 20 weeks) as a predictor as well as a treatment (4 category) by time interaction, and a random intercept to control for repeated measures within individuals (results for 4 and 20 weeks not presented). Additional regression models tested for treatment effect modification by baseline healthy eating and gVLM. Specifically, the baseline values of the aHEI or the gVLM were included in the mixed effects model along with its interaction with the 4 categories of with treatment. To provide a more flexible relationship to be tested than simply linear associations, the aHEI and gVLM measures were trichotomized into low, medium, and high values based on tertiles. Post-hoc tests of the treatment effect within each tertile of aHEI and gVLMB were performed when the interaction test was found to be significant at 0.10.

Analyses were carried out using SAS 9.4. Cohen’s d effect sizes were calculated for all treatment effect to allow direct comparison of magnitude using the baseline standard deviation of each variable across all groups.

The planned sample size, i.e. 50 completers per treatment group, and 12 completers per treatment group in the MRI sub-study, was based on power calculations based on data from the previous study to ensure at least 80% power for the expected effects on the primary neurocognitive endpoint, i.e., the object-recognition task, and the secondary MRI endpoint, i.e., CBV. The expected effect size of high intake versus placebo, Cohen’s d = 0.72, for the object-recognition task, and Cohen’s d = 1.9 for CBV, were based on prior studies^8^.

### Data availablity

The datasets generated during and/or analysed during the current study are available from the corresponding author on reasonable request.

## Clinical trial registry

clinicaltrials.gov Identifier: NCT02312310; (https://clinicaltrials.gov/ct2/show/NCT02312310).

## Supplementary Information

Supplementary Information
