# Supplementary material for: Insights into the role of diet and dietary flavanols in cognitive aging: results of a randomized controlled trial
Source: Sci Rep. 2021 Feb 15;11:3837. doi: 10.1038/s41598-021-83370-2 (PMC7884710; doi:10.1038/s41598-021-83370-2)
Supplement: Supplementary file 1 — Supplementary Information [file 41598_2021_83370_MOESM1_ESM.pdf]

# SUPPLEMENTARY INFOMRATION

# Insights into the role of diet and dietary flavanols in cognitive aging: results of a randomized controlled trial

**Richard P. Sloan, PhD[1,2,*], Melanie Wall, PhD[2], Lok-Kin Yeung, PhD[3,4], Tianshu Feng, MS[2] , Xinyang Feng, PhD[3], Frank Provenzano, PhD[3,4], Hagen Schroeter, PhD[5] , Vincenzo Lauriola, MS[1], Adam M. Brickman, PhD[3,4] , Scott A. Small, MD[3,4] *.**

[1]Division of Behavioral Medicine, Department of Psychiatry, Columbia University Irving Medical Center, 622 West 168th St., New York NY 10032, USA ; [2]New York State Psychiatric Institute, 1050 Riverside Drive, New York NY 10032, USA; [3]Department of Neurology, Vagelos College of Physicians and Surgeons, Columbia University, 622 West 168th St., New York NY 10032, USA; [4]Taub Institute for Research on Alzheimer's Disease and the Aging Brain, Vagelos College of Physicians and Surgeons, Columbia University, 622 West 168th St., New York NY 10032; [5]Mars Inc., 6885 Elm St, McLean, VA 22101, United States; *Corresponding Authors

---

**Index**

Supplementary Figure 1 ________page 2

Supplementary Methods________page 3-4

    Neuroimaging __________page 3

    Test material description__page 4

# SUPPLEMENTARY FIGURE 1

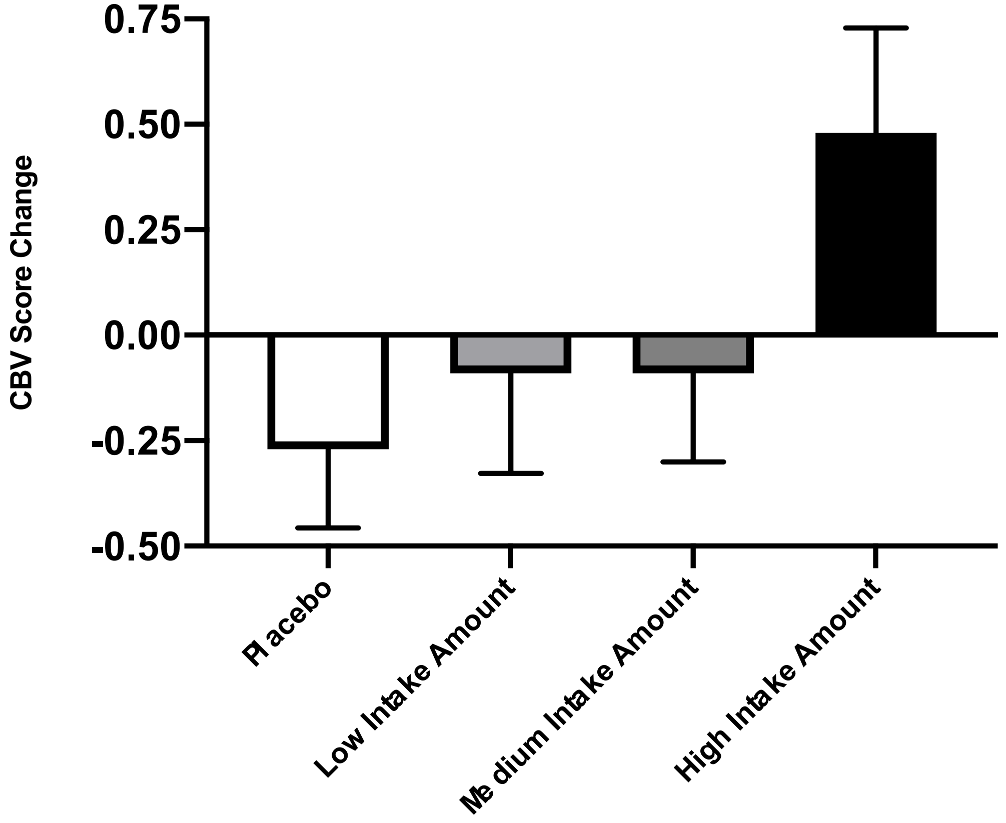


**Supplementary Figure 1**: Changes in CBV-fMRI (not adjusted for baseline) before and 12 weeks after daily intake of placebo and flavanols at a low (260 mg), medium (510 mg) and high (770 mg) intake level. Data are expressed as mean ± SD (not adjusted for baseline).

# SUPPLEMENTARY METHODS

## 1. NEUROIMAGING

### Exclusion Criteria

MRI-related exclusion criteria included cardiac pacemaker, internal pump, insulin pump, tattoo eyeliner, wire suture, internal metal objects, metal slivers in eye, prosthesis, hearing aid implants, neurostimulator, metal fragments, brain aneurysm clips, vascular clips, breast expander, vena cava filter, heart valve, metal stents, asthmatic symptoms within the past 3 years, sickle cell disease, kidney disease, pregnant, claustrophobic, wheelchair bound, machinist or ever worked with heavy metals and contraindication to gadolinium.

### MRI Acquisition

Subjects eligible for MRI scans received them at two times (weeks 0 and 12) on a GE Prisma 3.0T MRI scanner using similar acquisition criteria as previously published[1,2]. Eligibility for MRI scanning included eligibility criteria for injection of Dotarem contrast agent. To be eligible for scanning, all subjects must not have received any contrast injection prior and undergo a fingerstick creatinine measure to measure glomerular filtration rate (GFR) the day of the scanner which falls below 60ml/min. Briefly, subjects were under the supervision of a physician or nurse who placed an intravenous line and loaded an appropriate weight adjusted dose of Dotarem contrast agent (0.1 mg/kg) into an auto-injector. A structural TFE T1-weighted high resolution (1x1x1 $mm^3$) structural image was acquired for cortical and subcortical segmentation. A structural T2 GRE image was acquired in addition to aid in the hippocampal segmentation. Following that, a pair of TFE T1 weighted images were acquired (0.68x0.68x3$mm^3$) oblique to the long axis of the hippocampus, prior to and after the bolus injection of the contrast agent and a four-minute period of rest. Patients remained in the scanner during the entire procedure.

### MRI Processing

Whole brain CBV images were generated from the pair of pre and post-contrast MRI images as described previously[1]. In short, co-registered pre-contrast images were subtracted from post-contrast images and divided by a value of pure blood signal obtained from an automatic segmentation of the superior sagittal sinus. Structural images were analyzed through FreeSurfer 6.0[3] using both T1 and T2 images. The hippocampal subfield segmentation algorithm[4] was applied to images with a trained rater editing images. The CA4-DG-ML regions were merged for each participant's hippocampus bilaterally to capture the region of the DG, and further restricted from the 3$^{rd}$ slice anteriorly, and 4$^{th}$ slice posteriorly to localize the body of the hippocampus. Weighted mean values for all DG body ROIs were obtained after a filter was previously applied to exclude the effects of epicortical and high signal reflective of pure vasculature.


1. Brickman AM, Khan UA, Provenzano FA, et al. Enhancing dentate gyrus function with dietary flavanols improves cognition in older adults. *Nat Neurosci.* 2014;17(12):1798-1803.
2. Khan UA, Liu L, Provenzano FA, et al. Molecular drivers and cortical spread of lateral entorhinal cortex dysfunction in preclinical Alzheimer's disease. *Nat Neurosci.* 2014;17(2):304-311.
3. Fischl B. FreeSurfer. *Neuroimage.* 2012;62(2):774-781.
4. Iglesias JE, Augustinack JC, Nguyen K, et al. A computational atlas of the hippocampal formation using ex vivo, ultra-high resolution MRI: Application to adaptive segmentation of in vivo MRI. *Neuroimage.* 2015;115:117-137.

## 2. TEST MATERIAL DESCRIPTION

All test materials were provided by Mars, Inc. The vegetarian capsules either contained a placebo or varying levels of cocoa extract (Cocoapro®-processed cocoa extract) formulated to deliver 0 mg, 260 mg, 510 mg or 770 mg of cocoa flavanols per 4-capsule serving. The total amount of cocoa flavanols refrenced here is defined as the sum of all monomeric flavanols and their oligomers (i.e., procyanidins) with a degree of polymerization up to and including 7 (i.e. DP 1-7). The total cocoa flavanol content and flavanol stereoisomers were determined by HPLC with fluorescence detection in accordance with Bussy et al. [1] and Machonis et al. [2], respectively. Placebo capsules contained biologically inert materials. Detailed information of the test material composition are provided in **Table A.** All capsules were indistingusiable in appearance. Capsules were provided in 120-count (30-d supply) bottles, labeled with an alphanumeric code. All participants and all researchers involved in the execution of the study remined masked with regard to the designation of the test material until post-study database lock and formal unmasking.

**Table A.** Content of flavanol and methylxanthines in test material. Serving size consisted of 4 capsules per intake. Data are expressed as mean ± SD of the amount of flavanols and methylxanthies per 4 capsule serving.

| | **Placebo** | **Low Intake Amount** | **Medium Intake Amount** | **High Intake Amount** |
|---|---|---|---|---|
| Total Cocoa Flavanol Content (DP 1-7), mg | 0 | 260 ± 10 | 510 ± 20 | 770 ± 30 |
| (-)-epicatechin, mg | 0 | 45 ± 5 | 90 ± 10 | 135 ± 15 |
| (-)-catechin, mg | 0 | 15 ± 1 | 25 ± 2 | 40 ± 3 |
| (+)-catechin, mg | 0 | 2 ± 1 | 4 ± 1 | 5 ± 1 |
| (+)-epicatechin, mg | 0 | n.d. | n.d. | n.d. |
| Caffeine, mg | 0 | 8 ± 1 | 15 ± 1 | 25 ± 2 |
| Theobromine, mg | 0 | 30 ± 2 | 55 ± 3 | 80 ± 5 |

1. Bussy U, May BR, Olanrewaju Y, et al. Reliable, accessible and transferable method for the quantification of flavanols and procyanidins in foodstuffs and dietary supplements. *Food & Function.* 2020.

2 Machonis P, Jones M, Schaneberg B, Kwik-Uribe C, Dowell D. Method for the determination of catechin and epicatechin enantiomers in cocoa-based ingredients and products by high-performance liquid chromatography: First Action 2013.04 *J AOAC Int.* 2014 Mar-Apr;97(2):506-9.
